# Supplementary material for: Causal Relationship between Aging and Anorexia Nervosa: A White-Matter-Microstructure-Mediated Mendelian Randomization Analysis
Source: Biomedicines. 2024 Aug 16;12(8):1874. doi: 10.3390/biomedicines12081874 (PMC11351342; doi:10.3390/biomedicines12081874)
Supplement: Supplementary file 1 [file biomedicines-12-01874-s001.zip › Supplementary Figure/Figure S8/Leaveoneout plot of MR analysis for TL to ubm-b-2105.pdf]

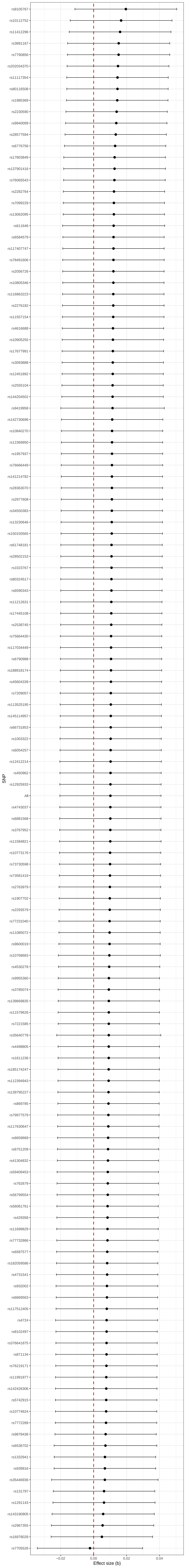
rs8105767
rs10112752
rs11412296
rs3891167
rs7790856
rs202034370
rs11117354
rs80116508
rs1985369
rs2230590
rs9940099
rs28577594
rs6776756
rs17803849
rs137901416
rs76065543
rs2282764
rs7099229
rs13062095
rs611646
rs6584579
rs117407747
rs78491606
rs2056726
rs10805346
rs116863223
rs2276182
rs11557154
rs4616688
rs10905255
rs17677991
rs3093888
rs12451892
rs2555104
rs144204502
rs9419958
rs142730696
rs10840270
rs12369950
rs1957937
rs76666449
rs141214782
rs28363070
rs2977608
rs34550383
rs13230646
rs150150565
rs61748181
rs28502153
rs1023767
rs80324517
rs6590343
rs11212631
rs17445108
rs2538745
rs75664430
rs117034449
rs6790988
rs188918174
rs45604339
rs7209057
rs113525195
rs145114957
rs66731853
rs1003322
rs6054257
rs12412214
rs450962
rs12925933
All
rs4743037
rs6881568
rs3767952
rs11584821
rs10773176
rs73730598
rs73581419
rs2763979
rs1907702
rs2293579
rs77231040
rs11085072
rs9600019
rs10768683
rs4530278
rs9955360
rs3785074
rs139669835
rs11579626
rs7221585
rs35640778
rs4498805
rs1611236
rs185174247
rs112394943
rs139795227
rs869785
rs79977579
rs117630647
rs6659669
rs6751209
rs41304832
rs59409453
rs762679
rs56799554
rs56061761
rs429358
rs11699829
rs77732866
rs6587577
rs182059586
rs4731541
rs932002
rs6669563
rs117512405
rs4724
rs8102497
rs376641875
rs871134
rs76219171
rs11991877
rs142426306
rs5742915
rs10774624
rs7772289
rs9878436
rs6536702
rs1332941
rs939916
rs35446936
rs131797
rs1291143
rs143190905
rs2967355
rs16978028
rs7705526
SNP
−0.02
0.00
0.02
0.04
Effect size (b)
